# Supplementary material for: The Interplay of Inter- and Intramolecular Hydrogen Bonding in Ether Alcohols Related to n-Octanol
Source: Molecules. 2025 Jun 4;30(11):2456. doi: 10.3390/molecules30112456 (PMC12156905; doi:10.3390/molecules30112456)
Supplement: Supplementary file 1 [file molecules-30-02456-s001.zip › SI-MD-etheralcohols.pdf]

The Interplay of Inter- and Intramolecular Hydrogen Bonding in Ether Alcohols Related to *n*-Octanol

Markus M. Hoffmann,*[1] Troy Smith,[1] and Gerd Buntkowsky*[2]

[1] *Department of Chemistry and Biochemistry, State University of New York Brockport, Brockport, NY, 14420, USA*

[2] *Institute of Physical Chemistry, Technical University Darmstadt, Peter-Grünberg-Straße 8, D-64287 Darmstadt, Germany*

Corresponding authors: mhoffman@brockport.edu
gerd.buntkowsky@chemie.tu-darmstadt.de

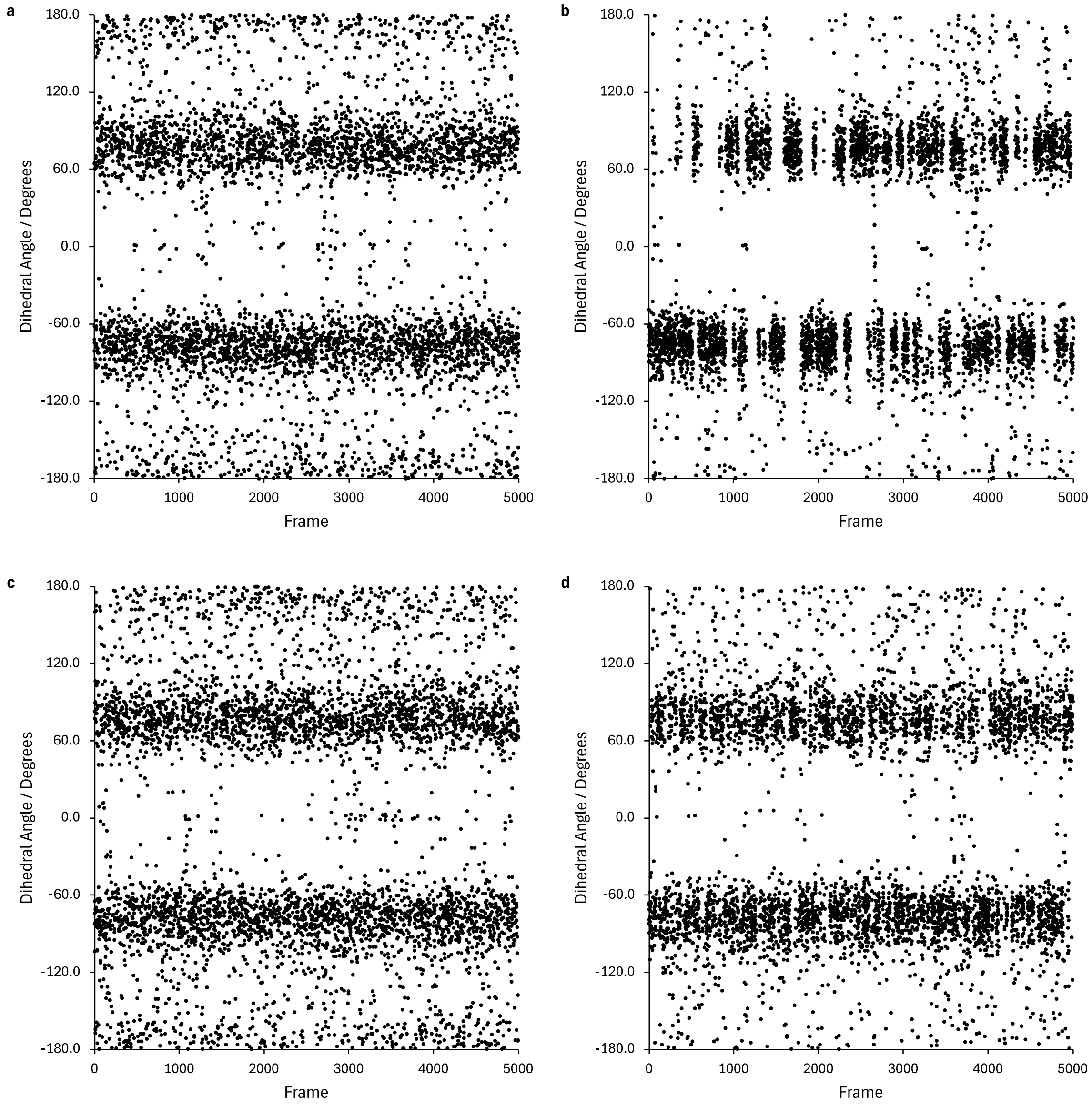

Figure S1. HO-CH_2-CH_2-OR dihedral angle of a single molecule of 1-hexoxymethanol found in each of 5000 frames of accumulated simulating at 298 K with (a) CHARMM and (b) OPLS, and at 358 K with (c) CHARMM and (d) OPLS. In CHARMM simulations (Figure S1a, Figure S1**Figure S1**c), dihedrals frequently transition as the model inherently allows sampling of broader conformational configurations. OPLS simulations (Figure S1b, Figure S1d) show more rigid dihedrals with infrequent transitions constrained by energy barriers in the functional form. Temperature increases minimally impact CHARMM's flexibility but enhances OPLS dihedral transitions, overcoming imposed energy barriers.

Table S1. Simulated and Experimental Densities, ρ, in Units of $kg\cdot m^{-3}$

Force Field	OPLS	CHARMM	Exp.	OPLS	CHARMM	Exp.
alcohol	298K			358K		
n-octanol	824.0	820.0	821.7	764.2	764.3	778.0
1-hexoxymethanol	897.2	874.5		836.7	812.4	
2-pentxoxyethanol	882.0	880.7	907.4	820.3	817.6	856.8
3-butoxypropanol	899.5	882.9	892.8	839.4	821.0	844.2
4-propoxybutanol	899.6	888.3	893.4	838.4	828.3	846.0
5-ethoxypentanol	910.5	894.5	898.2	849.8	836.5	851.8
6-methoxyhexanol	911.9	897.6	914.4	853.8	840.9	868.7

Table S2. Simulated and Experimental Viscosities, η, in Units of $mPa\cdot s$

Force Field	OPLS	CHARMM	Exp.	OPLS	CHARMM	Exp.
alcohol	298K			358K		
n-octanol	7.74	12.30	7.67	1.10	1.45	1.45
1-hexoxymethanol	8.20	5.32		1.40	1.10	
2-pentxoxyethanol	4.30	5.01	4.06	1.20	1.20	1.08
3-butoxypropanol	14.31	9.30	4.95	1.63	1.25	1.21
4-propoxybutanol	11.06	10.20	5.70	1.70	1.50	1.35
5-ethoxypentanol	17.37	12.20	6.12	1.70	1.80	1.46
6-methoxyhexanol	11.83	12.51	6.68	1.55	1.90	1.56

Table S3. Simulated and Experimental Self-Diffusion, *D*, Coefficients in Units of $10^{-11}\cdot m^2\cdot s^{-1}$

Force Field	OPLS	CHARMM	Exp.	OPLS	CHARMM	Exp.
alcohol	298K			358K		
n-octanol	16.6	8.7	14.8	121.2	83.2	84.6
1-hexoxymethanol	13.5	21.4		99.2	128.7	
2-pentxoxyethanol	24.8	18.7	29.3	120.0	111.4	119.5
3-butoxypropanol	8.0	12.9	22.2	76.4	96.2	99.5
4-propoxybutanol	10.6	10.5	20.2	88.6	83.1	99.3
5-ethoxypentanol	8.8	8.2	19.3	78.2	70.2	87.7
6-methoxyhexanol	11.0	10.0	18.3	83.2	75.7	84.2

Table S4. Simulated and Experimental Isobaric Heat Capacities, C_p, in Units of $J \cdot K^{-1} \cdot mol^{-1}$

Force Field	OPLS	CHARMM	Exp.	OPLS	CHARMM	Exp.
alcohol	298K			358K		
n-octanol	725.5	632.4	304.4	663.7	665.9	376.4
1-hexoxymethanol	632.6	630.1		665.1	622.2	
2-pentxoxyethanol	622.6	659.9	302.3	592.9	629.2	340.5
3-butoxypropanol	642.4	667.9	300.3	625.3	617.2	340.8
4-propoxybutanol	632.6	616.2	292.3	599.8	618.6	335.9
5-ethoxypentanol	654.1	618.5	294.3	656.6	630.7	339.4
6-methoxyhexanol	635.8	633.9	296.0	641.3	601.1	334.3

Table S5. Simulated and Experimental Values for ($D\ \eta\ T^{-1}$) in Units of $10^{-14}\ N \cdot s^{-1} \cdot K^{-1}$

Force Field	OPLS	CHARMM	Exp.	OPLS	CHARMM	Exp.
alcohol	298K			358K		
n-octanol	0.43	0.36	0.38	0.37	0.34	0.34
1-hexoxymethanol	0.37	0.38		0.39	0.40	
2-pentxoxyethanol	0.36	0.31	0.40	0.40	0.37	0.36
3-butoxypropanol	0.38	0.40	0.37	0.35	0.34	0.34
4-propoxybutanol	0.39	0.36	0.39	0.42	0.35	0.38
5-ethoxypentanol	0.51	0.34	0.40	0.37	0.35	0.36
6-methoxyhexanol	0.44	0.42	0.41	0.36	0.40	0.37

Table S6. Arrhenius Activation Energies in Units of $kJ \cdot mol^{-1}$

Force Field	OPLS	CHARMM	Exp.	OPLS	CHARMM	Exp.
alcohol	Self-Diffusion			Viscosity		
n-octanol	29.4	33.3	25.8	28.8	31.6	24.6
1-hexoxymethanol	29.5	26.6		26.1	23.3	
2-pentxoxyethanol	23.3	26.4	20.8	18.9	21.1	19.5
3-butoxypropanol	33.4	29.7	22.2	32.2	29.7	20.8
4-propoxybutanol	31.5	30.6	23.5	27.7	28.3	21.2
5-ethoxypentanol	32.4	31.7	22.4	34.4	28.3	21.2
6-methoxyhexanol	29.9	29.9	22.6	30.0	27.9	21.5

Table S7. Exponential Fit Parameters and Statistics for Complementary Cumulative Distribution Function.

	Inter,OH-OH			Inter, OH-OE			Intra, OH-OE		
n	*C*	γ / ps^{-1}	R^2	*C*	γ / ps^{-1}	R^2	*C*	γ / ps^{-1}	R^2
				CHARMM, 298 K					
0	0.926	0.059	0.993	-	-	-	-	-	-
1	1.320	0.320	0.995	0.838	0.066	0.972	-	-	-
2	1.119	0.170	0.997	1.469	0.406	0.998	-	-	-
3	0.958	0.078	0.994	1.114	0.205	0.987	1.052	0.016	0.994
4	0.956	0.073	0.994	1.041	0.151	0.989	0.848	0.015	0.982
5	0.925	0.064	0.993	0.995	0.116	0.990	1.098	0.231	0.953
6	0.955	0.075	0.994	1.066	0.157	0.992	1.908	0.651	0.997
				OPLS-AA, 298 K					
0	1.076	0.137	0.997	-	-	-	-	-	-
1	1.195	0.234	0.995	0.984	0.102	0.992	-	-	-
2	1.146	0.219	0.990	1.776	0.598	0.994	-	-	-
3	0.984	0.089	0.995	1.032	0.138	0.991	1.033	0.063	0.998
4	1.006	0.100	0.995	1.159	0.222	0.992	0.988	0.030	0.999
5	1.021	0.111	0.995	1.092	0.172	0.993	1.353	0.360	0.987
6	1.056	0.126	0.997	1.174	0.216	0.996	3.187	1.167	0.987
				CHARMM, 358 K					
0	1.593	0.476	0.999	-	-	-	-	-	-
1	4.024	1.393	1.000	1.571	0.481	0.995	-	-	-
2	2.406	0.879	1.000	4.016	1.391	1.000	-	-	-
3	1.786	0.587	1.000	3.193	1.163	1.000	1.312	0.025	0.926
4	1.733	0.556	1.000	2.595	0.956	1.000	1.122	0.032	0.986
5	1.662	0.517	0.999	2.297	0.835	1.000	1.734	0.553	1.000
6	1.729	0.554	1.000	2.492	0.915	1.000	4.042	1.398	0.998
				OPLS-AA, 358 K					
0	2.632	0.969	1.000	-	-	-	-	-	-
1	2.179	0.781	1.000	1.469	0.406	0.998	-	-	-
2	1.985	0.692	0.999	4.027	1.394	1.000	-	-	-
3	1.963	0.678	1.000	2.646	0.974	1.000	1.311	0.083	0.948
4	2.136	0.761	1.000	3.755	1.323	1.000	1.419	0.039	0.916
5	2.395	0.875	1.000	3.201	1.164	1.000	1.734	0.553	0.989
6	2.407	0.880	1.000	3.411	1.227	1.000	6.189	1.823	1.000
